# Immune checkpoint inhibitor‐induced myasthenia gravis, myocarditis, and myositis: A case report

**DOI:** 10.1002/ccr3.8968

**Published:** 2024-06-10

**Authors:** Arjun Basnet, Nava Raj Sharma, Sudarshan Gautam, Saral Lamichhane, Sajog Kansakar, Kripa Tiwari, Madalasa Pokhrel, Sehajpreet Singh

**Affiliations:** ^1^ Maimonides Medical Center Brooklyn New York USA; ^2^ Manipal College of Medical Sciences Pokhara Nepal; ^3^ Gandaki Medical College Pokhara Nepal; ^4^ Montefiore New Rochelle Hospital New Rochelle New York USA

**Keywords:** immune checkpoint inhibitors, myasthenia, myocarditis, myositis

## Abstract

**Key Clinical Message:**

Immune checkpoint inhibitors can rarely lead to occurrence of myositis, myocarditis, and myasthenia gravis (MG). Early recognition and multidisciplinary management are crucial for optimal outcomes. Vigilance for overlapping toxicities is essential in patients receiving combination immunotherapy.

**Abstract:**

The use of immune checkpoint inhibitors (ICIs) has revolutionized cancer treatment, but it is associated with immune‐related adverse events (IRAEs) affecting various organ systems. The simultaneous occurrence of MG, myocarditis, and myositis highlights the complex nature of IRAEs. Early recognition and comprehensive multidisciplinary management are crucial for optimal patient outcomes. We present a unique case report of a 76‐year‐old male patient with advanced melanoma who developed concurrent myositis, myocarditis, and MG while receiving combination immunotherapy with Nivolumab and Ipilimumab. This case underscores the significance of recognizing and addressing the “Terrible Triad” of IRAEs in patients receiving ICIs. Healthcare providers should maintain a high index of suspicion for overlapping toxicities and promptly initiate appropriate interventions.

## 1 INTRODUCTION

Immune checkpoint inhibitors (ICIs) have significantly advanced cancer treatment but can lead to rare and severe immune‐related adverse events (IRAEs). Various incidences of myositis, myocarditis, and neurological manifestations, including polyneuropathy and myasthenia gravis (MG), have been reported as IRAEs associated with ICIs. Previous studies have documented a high incidence of concurrent myositis and MG in approximately 30%–40% and 10% of patients with immune‐related myocarditis, respectively.^1^ The occurrence of myocarditis associated with ICIs is not well‐established, potentially due to limited screening in early studies. Recent reports indicate an incidence of 0.27%–1.14% for ICI‐associated myocarditis.^1^, ^2^ Additionally, MG, a rare neuromuscular disorder, is rarely observed in patients treated with ICIs, especially PD‐1 inhibitors, with an incidence ranging from 0.12%–0.2%.^3^ The combination of anti‐CTLA‐4 (Cytotoxic T‐Lymphocyte‐Associated Antigen 4) and anti‐PD‐1 (Programmed Death 1) therapies has been identified as a significant risk factor for this terrible triad of MG, Myocarditis, and Myositis, with ipilimumab and nivolumab combination therapy carrying a 4.74‐fold higher risk compared to nivolumab alone.^2^ Here, we present a unique case report of a 76‐year‐old male patient with advanced melanoma who developed concurrent myositis, myocarditis, and MG while receiving combination immunotherapy with Nivolumab and Ipilimumab.

## 2 CASE HISTORY/EXAMINATION

A 76‐year‐old male patient with a medical history of coronary artery disease, treated with percutaneous intervention (PCI) and stent placement 13 years ago, as well as hypertension, hyperlipidemia, diabetes, hyperthyroidism (managed with partial thyroidectomy), and a previous history of melanoma treated with excision and immunotherapy, presented with a progressive onset of shortness of breath over a period of 6 days. The patient had received his most recent dose of immunotherapy, comprising 100 mg of Ipilimumab and 300 mg of Nivolumab, 3 weeks prior to his presentation. Initially, the shortness of breath occurred during exertion but gradually worsened to affect routine daily activities. The patient also experienced weakness in the facial muscles but denied symptoms such as difficulty breathing when lying down (orthopnea), sudden episodes of breathing difficulty at night (paroxysmal nocturnal dyspnea), chest pain, or dizziness.

Upon arrival at the emergency department, the patient's vital signs were stable. A physical examination revealed mild weakness in the facial muscles, while the remainder of the examination did not yield any significant findings.

## 3 METHODS (INVESTIGATIONS AND TREATMENT)

**FIGURE 1:**
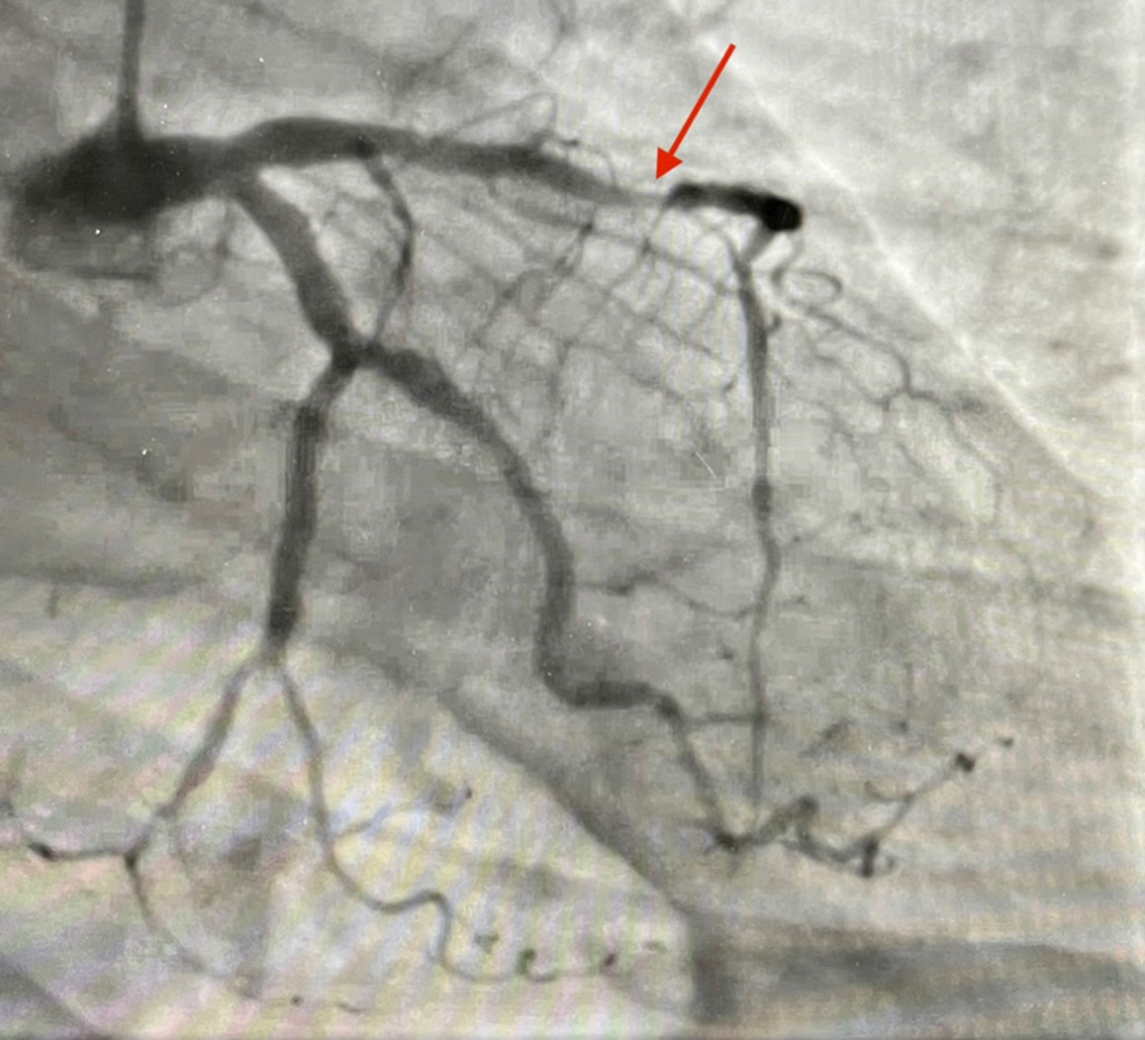
Mid‐LAD showing 99 percent stenosis.

Laboratory tests showed a rise in troponin levels from an initial value of 3.74 ng/mL–5 ng/mL, along with elevated liver enzymes, myoglobin, creatinine, and white blood cell count as summarized in Table 1. However, the electrocardiogram did not reveal any ST‐segment or T‐wave changes as in Figure 1. Additionally, the computed tomography angiography of the chest did not detect a pulmonary embolism or parenchymal lung disease. A bedside echocardiogram revealed preserved left ventricular ejection fraction without significant wall motion abnormalities or valvular heart disease. Serial electrocardiograms did not display significant changes compared to the admission electrocardiogram.

**TABLE 1:** Initial laboratory values.

Tests	Result	Reference range
Blood glucose	216 mg/dL	70–130 mg/dL (fasting)
TSH, serum (thyroid stimulating hormone)	2.56 mCIU/mL	0.4–4.5 mCIU/mL
T4 Free, serum (free thyroxine)	0.78 ng/dL	0.7–1.8 ng/dL
T3 Total, serum (total triiodothyronine)	0.63 ng/mL	0.8–2.0 ng/mL
International normalized ratio	INR: 0.9	0.8–1.2
Prothrombin time	10.4 sec	11–13 s
Phosphorus, serum	6.4 mg/dL	2.5–4.5 mg/dL
Magnesium, serum	2.1 mg/dL	1.7–2.3 mg/dL
Hepatitis C antibody	Nonreactive	‐
Hemoglobin A1C, plasma	7.0%	Less than 5.7% (nondiabetic)
D‐Dimer assay, plasma	269 DDU ng/mL	Less than 500 DDU ng/mL
CKMB battery (creatine kinase MB)	CPK, serum: 6235 IU/L	40–110 IU/L
CKMB	228.8 ng/mL	Less than 3 ng/mL
Cardiac troponin I‐CtNI	3.74 ng/mL	Less than 0.04 ng/mL
B‐type natriuretic peptide	31 pg/mL	Less than 100 pg/mL
Activated partial thromboplastin time	59.3 s	25–35 s
Creatinine, serum	1.1 mg/dL	0.6–1.3 mg/dL
Albumin, serum	3.5 g/dL	3.5–5.0 g/dL
Alkaline phosphatase	60 IU/L	40–129 IU/L
ALT, serum	249 IU/L	0–44 IU/L
AST, serum	345 IU/L	0–40 IU/L
Bilirubin, direct	0.1 mg/dL	0–0.3 mg/dL
Bilirubin, total	0.4 mg/dL	0–1.2 mg/dL
Total Protein, serum	6.5 g/dL	6.0–8.3 g/dL

The patient was initiated on a heparin drip due to persistent shortness of breath and elevated troponin levels. Subsequent coronary angiography revealed 99 percent stenosis in the mid‐left anterior descending artery (LAD) as in Figure 1, for which percutaneous transluminal coronary angioplasty, shockwave lithotripsy, and placement of a drug‐eluting stent were performed. The patient's dyspnea did not improve despite coronary stenting; troponin levels remained elevated, likely due to myocarditis. While in the hospital, the patient developed diffuse weakness, most notably in the extra ocular, facial, nasopharyngeal, neck flexor, respiratory muscles, and all four extremities, with proximal weakness being more pronounced. Orientation, superficial sensation, and coordination of the upper extremities remained largely intact.

The patient's computed tomography angiography of the chest revealed vocal cord paralysis. Consultation with neurology and oncology services suggested IRAEs from checkpoint inhibitors, possibly leading to myasthenia crisis and myocarditis. Positive tests for MG‐specific antibodies confirmed the suspicion, while lumbar puncture ruled out other conditions.

## 4 RESULT AND OUTCOME

Respiratory distress worsened despite increased oxygen support, leading to elective intubation for airway protection. Empirical treatment with methylprednisolone was initiated on the fourth day of hospitalization. Recurrent wide‐complex tachycardia was unresponsive to cardioversion and adenosine. The patient received amiodarone, lidocaine, and later procainamide. Renal and liver function deteriorated, requiring continuous venovenous hemodiafiltration (CVVHDF). Despite resuscitative efforts, the patient experienced multiple cardiac arrests and could not be revived and died on the 13th day of admission.

## 5 DISCUSSION

ICIs are a class of monoclonal antibodies that target specific inhibitory receptors in the immune system, namely CTLA‐4, PD‐1, and PD‐L1 (programmed cell death ligand 1).^4^ These receptors are predominantly found on T cells, a type of immune cell. By blocking the interaction between these receptors, ICIs enhance the immune response against cancer cells, thereby aiding in their elimination. Ipilimumab, approved in 2011, was the first immune‐related therapy to be authorized for the treatment of melanoma, followed by approvals for renal cell carcinoma and colorectal cancer.^5^ Since then, additional ICIs have been approved for various malignancies.

IRAEs are associated with disrupted T‐cell tolerance, increased levels of preexisting autoantibodies, and elevated inflammatory cytokines. This aberration leads to the activation of T‐cells, which mistakenly target healthy tissues, resulting in the development of IRAEs.^6^ Although the precise cause of myocarditis associated with ICIs remains uncertain, it is hypothesized that there may be shared antigens between tumors and cardiac tissue contributing to this immune reaction.^7^ The medical literature reports several other IRAEs, such as hepatitis, myositis, colitis, thyroiditis, MG, nephritis, pneumonitis, rhabdomyolysis, hypophysitis, neuritis, polyneuropathy, conjunctivitis/uveitis, syndrome of inappropriate antidiuretic hormone, encephalitis, and Stevens‐Johnson syndrome.^8^ In our case, the patient exhibited clinical manifestations suggestive of myositis, myocarditis, MG, and nephritis.

Our case report highlights the significant impact of the “Terrible Triad”—immune checkpoint inhibitor‐induced MG, myocarditis, and myositis. Some studies found that patients with all three toxicities had a notably higher risk of death than those with MG alone, MG and myositis, or myocarditis alone.^2^ Additionally, MG tended to manifest earlier than other neurotoxicities after immunotherapy. Interestingly, a separate study on ICI‐related myocarditis revealed that myositis and MG were the most common coexisting IRAEs.^1^ It is worth noting that the diagnosis of ICI‐related myositis can be challenging due to oculomotor weakness, which may complicate the identification of MG.^9^

ICI‐related myocarditis is a relatively rare but potentially fatal complication, with reported incidence rates ranging from 0.04% to 1.14%. Its mortality rate is significantly high, ranging from 25% to 50%.^10^ Combination therapy with ICIs increases the risk of developing myocarditis and the associated mortality. The clinical presentation of myocarditis can vary. Severe cases may present with life‐threatening symptoms such as cardiogenic shock and severe arrhythmias.

On the other hand, milder cases can resemble viral myocarditis, exhibiting symptoms similar to acute coronary syndrome, new‐onset heart failure, or chronic heart failure. Patients may also present with pericardial effusion, with or without pericarditis. It is crucial to differentiate myocarditis from other conditions like coronary artery disease (CAD) or other causes of heart failure, especially considering that cancer patients often have underlying CAD. Diagnostic tests are essential to confirm and manage suspected ICI cardiotoxicity. The probability of ICI‐associated myocarditis increases when patients present with concomitant IRAEs, particularly MG and myositis.^11^

American Society of Clinical Oncology (ASCO) has developed guidelines for managing IRAEs, including myocarditis. These guidelines categorize myocarditis into four severity grades,^1^, ^2^, ^3^, ^4^ with Grades 1 representing mild cases and 4 indicating the most severe cases.^6^ According to the guidelines, patients with suspected myocarditis should undergo a comprehensive evaluation, including cardiac biomarkers, electrocardiogram (ECG), chest x‐ray, echocardiogram, and cardiac MRI. While these tests provide valuable information, an endomyocardial biopsy is the gold standard diagnostic test for myocarditis.^12^ Invasive coronary angiography may also rule out coronary artery disease in patients with elevated troponin levels. When immune‐related myocarditis is suspected, it is crucial to promptly discontinue ICIs and initiate high‐dose glucocorticoid therapy. The ASCO clinical practice guidelines recommend a steroid tapering regimen over 4–6 weeks, although the optimal duration for tapering remains uncertain.^6^ In cases where glucocorticoids alone are insufficient, alternative immunomodulatory treatments such as intravenous immunoglobulin, mycophenolate, infliximab, anti‐thymocyte globulin, plasmapheresis, alemtuzumab, or abatacept may be considered.^7^ It is important to note that the management of immune‐related myocarditis should be tailored to each patient and their specific clinical circumstances.

## 6 CONCLUSION

Prompt recognition and discontinuation of the ICI, along with the use of high‐dose glucocorticoids, were crucial in managing the IRAEs. The response to treatment varied among the different conditions, highlighting the need for individualized approaches. This case report underscores the importance of early detection and intervention in IRAEs associated with ICIs. Treatment decisions should be made in collaboration with a multidisciplinary team, including oncologists, cardiologists, and other relevant specialists. Continued research is necessary to enhance our understanding and management of these complex immune‐related complications.

## AUTHOR CONTRIBUTIONS

**Arjun Basnet:** Conceptualization; methodology; writing – original draft. **Nava Raj Sharma:** Conceptualization; methodology; writing – original draft; writing – review and editing. **Sudarshan Gautam:** Conceptualization; methodology; writing – original draft. **Saral Lamichhane:** Conceptualization; methodology; writing – original draft; writing – review and editing. **Sajog Kansakar:** Conceptualization; methodology; writing – original draft. **Kripa Tiwari:** Conceptualization; methodology; writing – original draft. **Madalasa Pokhrel:** Conceptualization; methodology; writing – original draft. **Sehajpreet Singh:** Methodology; writing – review and editing.

## FUNDING INFORMTION

None.

## CONFLICT OF INTEREST STATEMENT

None.

## ETHICS STATEMENT

Not required.

## CONSENT

Written consent was taken from the patient and is available on request by the journal.

## Data Availability

All data regarding this case has been reported in the manuscript. Please contact the corresponding author if you are interested in any further information.

## References

[1] Moslehi JJ , Salem JE , Sosman JA , Lebrun‐Vignes B , Johnson DB . Increased reporting of fatal immune checkpoint inhibitor‐associated myocarditis. Lancet. 2018;391(10124):933. doi:10.1016/S0140-6736(18)30533-6 PMC6668330 29536852

[2] Johnson DB , Balko JM , Compton ML , et al. Fulminant myocarditis with combination immune checkpoint blockade. New Eng J Med. 2016;375(18):1749‐1755. doi:10.1056/NEJMoa1609214 27806233 PMC5247797

[3] Kao JC , Brickshawana A , Liewluck T . Neuromuscular complications of programmed cell Death‐1 (PD‐1) inhibitors. Curr Neurol Neurosci Rep. 2018;18(10):63. doi:10.1007/s11910-018-0878-7 30078154

[4] Wojtukiewicz MZ , Rek MM , Karpowicz K , et al. Inhibitors of immune checkpoints—PD‐1, PD‐L1, CTLA‐4—new opportunities for cancer patients and a new challenge for internists and general practitioners. Cancer Metastasis Rev. 2021;40(3):949‐982. doi:10.1007/s10555-021-09976-0 34236546 PMC8556173

[5] Wolchok JD , Hodi FS , Weber JS , et al. Development of ipilimumab: a novel immunotherapeutic approach for the treatment of advanced melanoma. Ann N Y Acad Sci. 2013;1291(1):1‐13. doi:10.1111/nyas.12180 23772560 PMC3910157

[6] Brahmer JR , Lacchetti C , Schneider BJ , et al. Management of Immune‐Related Adverse Events in patients treated with immune checkpoint inhibitor therapy: American Society of Clinical Oncology clinical practice guideline. J Clin Oncol. 2018;36(17):1714‐1768. doi:10.1200/JCO.2017.77.6385 29442540 PMC6481621

[7] Palaskas N , Lopez‐Mattei J , Durand JB , Iliescu C , Deswal A . Immune checkpoint inhibitor myocarditis: pathophysiological characteristics, diagnosis, and treatment. J Am Heart Assoc. 2020;9(2):e013757. doi:10.1161/JAHA.119.013757 31960755 PMC7033840

[8] Matzen E , Bartels LE , Løgstrup B , Horskær S , Stilling C , Donskov F . Immune checkpoint inhibitor‐induced myocarditis in cancer patients: a case report and review of reported cases. Cardio‐Oncol. 2021;7(1):27. doi:10.1186/s40959-021-00114-x PMC8351114 34365980

[9] Touat M , Maisonobe T , Knauss S , et al. Immune checkpoint inhibitor‐related myositis and myocarditis in patients with cancer. Neurology. 2018;91(10):e985‐e994. doi:10.1212/WNL.0000000000006124 30089619

[10] Salem JE , Manouchehri A , Moey M , et al. Cardiovascular toxicities associated with immune checkpoint inhibitors: an observational, retrospective, pharmacovigilance study. Lancet Oncol. 2018;19(12):1579‐1589. doi:10.1016/S1470-2045(18)30608-9 30442497 PMC6287923

[11] Pathak R , Katel A , Massarelli E , Villaflor VM , Sun V , Salgia R . Immune checkpoint inhibitor–induced myocarditis with myositis/myasthenia gravis overlap syndrome: a systematic review of cases. Oncologist. 2021;26(12):1052‐1061. doi:10.1002/onco.13931 34378270 PMC8649039

[12] Rroku A , Kottwitz J , Heidecker B . Update on myocarditis – what we know so far and where we may be heading. Eur Heart J Acute Cardiovasc Care. 2021;10(4):455‐467. doi:10.1177/2048872620910109 32319308

